# Surgical Management of Sacroiliac Joint Dislocations and Crescent Fractures: A Nine-Year Clinical Follow-Up

**DOI:** 10.3390/jcm14207139

**Published:** 2025-10-10

**Authors:** Hüseyin Utku Özdeş, Muhammed Köroğlu, İdris Çoban, Ahmet Harma, Okan Aslantürk

**Affiliations:** 1 Orthopaedics and Traumatology Department, Turgut Özal Medical Center, İnönü University Medical School, Malatya 44280, Turkey; dr.utkuozdes@gmail.com (H.U.Ö.); m.koroglu91@gmail.com (M.K.); ahmetharma@gmail.com (A.H.); 2 Orthopedics and Traumatology Clinic, Viranşehir State Hospital, Sanliurfa 63700, Turkey; idriscoban21@hotmail.com

**Keywords:** sacroiliac, joint dislocation, pelvis, injury

## Abstract

**Background**: Pelvic injuries of the sacroiliac joint are unstable and require surgical intervention following high-energy trauma. In this study, we aimed to present the long-term clinical outcomes of patients with sacroiliac joint separation and sacroiliac fracture dislocation (crescent) injury. We compared the surgical interventions performed on the sacroiliac joint based on patient clinical data. **Methods**: By reviewing the records of 850 pelvic fractures treated in our clinic between 2000 and 2020, we identified 110 patients with sacroiliac joint injuries who were included in the study. The fractures were classified based on patient files and radiographs. The patients were categorized according to the surgical interventions performed on the sacroiliac joint into two groups: closed reduction with percutaneous iliosacral screws and open reduction with plates and screws. We further divided the patients who underwent open reduction and plate–screw fixation into anterior and posterior surgical approaches. Clinical outcomes were obtained by evaluating patients using a subjective pelvic scoring system. Additionally, complications observed after surgeries were investigated. **Results**: A total of 121 fractures from 110 patients were included in the study. Eleven of the patients had bilateral sacroiliac joint injuries, for which bilateral surgery was performed. The mean age of the patients at the time of injury was 35.15 years (range from 6 to 80 years). The mean follow-up period was 103.45 months (range from 16 to 253 months). According to the scoring system, the highest success rate was observed in plate–screw operations performed through the anterior approach to the sacroiliac joint, with excellent to good results in approximately 92% of patients. Both open reduction and internal fixation through the posterior approach and closed reduction and percutaneous iliosacral screw surgery yielded successful functional results, with no statistically significant difference between the methods (*p* = 0.880). Regarding complications, the most important problems were infections associated with plate–screw procedures using the posterior approach and neurologic injuries resulting from closed reduction screw surgery. **Conclusions**: Effective management of sacroiliac joint injuries requires surgical expertise and individualized treatment strategies. With appropriate technique and fixation, both open and closed surgical methods can achieve satisfactory anatomical reduction and functional outcomes. Although standardized treatment protocols may be developed, tailoring the approach to each patient is more important for optimal clinical success.

## 1. Introduction

Pelvic ring injuries are usually seen after high-energy trauma and are accompanied by additional orthopedic injuries and organ-system injuries [1]. In these injuries, maintaining the patient’s life should be the priority; afterward, the restoration of limb functions is essential [2,3]. In terms of these functions, the anatomical stabilization of the posterior structures is crucial in pelvic fractures; while non-surgical treatments may be appropriate for injuries that involve only the anterior structures, surgical treatment is prioritized in injuries that involve the sacroiliac joints [4]. In surgical treatment planning, it is essential to first understand and accurately identify the fracture. Although various classifications have been developed for pelvic fractures based on the mechanism of injury and the affected structures, in clinical settings, injuries are primarily defined by the Young–Burgess classification, such as antero-posterior, lateral compression, and vertical shear injuries [5,6]. The AO/OTA classification has also been developed based on the Tile classification and provides a more detailed description of the fracture [7]. A separate classification has been developed for crescent fractures, which describes the fracture dislocation of the sacroiliac joint and is part of lateral compression fractures [8]. These classifications guide the surgeon regarding the mechanism of injury and treatment. However, instant X-rays and computed tomography images, especially in the sacroiliac joints or pubic symphysis, can provide misleading information regarding the amount of diastasis. Stress radiographs are required for definitive information. While a wide separation may make decision-making clearer, determining instability is crucial for surgery.

Sacroiliac joint dislocations and crescent fractures are posterior pelvic injuries related to instability and are currently treated with surgical intervention. The aim of treatment is the anatomical reduction and maintenance of the posterior bone structure. Closed reduction, percutaneous cannulated iliosacral screws, and open reduction with screw and plate fixation methods are available. In the literature, middle-to-long-term results are limited. Thus, in this study, we aimed to compare the long-term clinical outcomes of patients with sacroiliac joint dislocations or crescent fractures, who were followed for nine years after surgical treatment, and to assess the impact of different surgical techniques on the functional results.

## 2. Methods

### 2.1. Patient Selection

Ethics committee approval was obtained from the Inonu University Scientific Research and Publication on 29 June 2021, with the number 2021/2237. A retrospective evaluation was conducted on 850 patients with pelvic injuries treated in the Department of Orthopedics and Traumatology between 2000 and 2020. Among these patients, 110 had sacroiliac joint dislocations and crescent fractures and were treated with iliosacral screws and/or plates and screws. Patients who underwent open surgery or closed percutaneous screw placement for sacroiliac joint dislocations and crescent fractures, those with complete radiological and medical records, and those with at least one year of follow-up were included in the study. Patients who were treated conservatively, patients with incomplete radiologic or medical records, patients with a follow-up period of less than 12 months, and patients with pelvic fractures without sacroiliac joint intervention were excluded from the study.

### 2.2. Evaluation and Treatment of Patients

The patient files were analyzed to obtain the physical examination findings of the patients at the time of injury. Pelvic radiographs taken after the injury, follow-up radiographs, and post-treatment radiographs were also evaluated. The radiographs were obtained from the X-ray film archive of the Department of Orthopaedics and Traumatology and the hospital PACS system archive.

Patients with sacroiliac joint separation and crescent fractures were initially evaluated in the emergency department. Emergency and orthopedic physicians conducted a detailed trauma examination and recorded the information. Pelvic AP radiographs were obtained as the first step for the patients. Inlet and outlet pelvic radiographs were taken for applicable patients. Computed tomography images were obtained to evaluate the posterior structures and fractures more clearly. Depending on the type of fracture and in patients with vital risk, pelvic bandaging or external fixation was applied as the first step for bleeding [9]. Isolated pelvic fractures without multiple traumas and hemodynamically stable patients were followed up in the Orthopedics and Traumatology Department. Follow-up for patients with multiple system injuries and for hemodynamically unstable patients was conducted in the intensive care unit. Surgery for hemodynamically stable patients with isolated pelvic fractures was performed within a few days after preoperative preparation. Operations for hemodynamically unstable patients were carried out after the patients were stabilized and the surgical contraindications were resolved.

The surgical technique was chosen based on the operating surgeon’s preference. For anterior pelvic injuries, external fixators, screws, or plate screws were applied, while iliac screws or plate screws were used for sacroiliac joint separations and crescent fractures. Intraoperative AP pelvic, inlet–outlet pelvic radiographs, and lateral radiographs were taken to evaluate the posterior structures. Initially, for each patient, closed reduction was performed under fluoroscopy for sacroiliac joint dislocations and fractures, and the reduced fracture and/or fractured dislocation were stabilized with one or two iliac screws. In cases where closed reduction could not be performed during intraoperative imaging, or where certainty about the reduction could not be established, the ilioinguinal approach was utilized to directly expose the sacroiliac joint, allowing for open reduction and internal fixation with a plate screw or just a screw. In selected cases of crescent fractures, patients were positioned prone, and a posterior approach to the joint was utilized, with internal fixation accomplished from the posterior side.

After surgery, the patients were monitored in the ward or intensive care unit based on the evaluations of the anesthesiologists. Postoperative bleeding was monitored, and neurological examinations were conducted and documented. Stable patients were seen in the outpatient clinic at 2 weeks, 6 weeks, and at 3, 6, and 12 months after discharge. Follow-up was then conducted annually.

**Figure 1:**
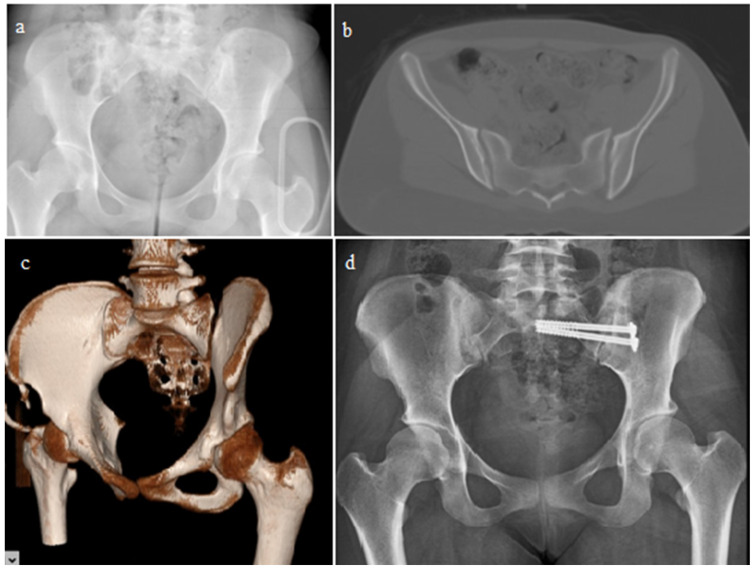
A 32-year-old woman with pure sacroiliac joint dislocation. Two screws were applied percutaneously. Pelvic radiography (**a**), tomography images of the pelvis (**b**,**c**), and postoperative third-year pelvic radiography (**d**).

**Figure 2:**
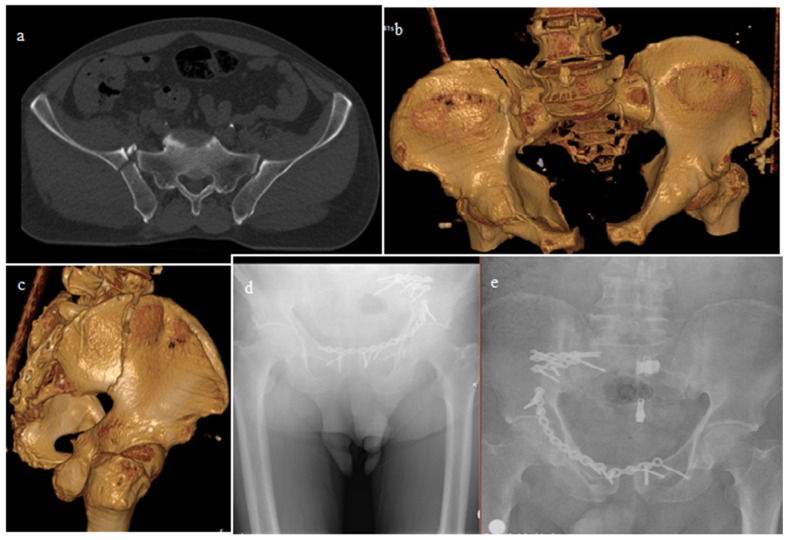
A 61-year-old male patient, experiencing a non-vehicle traffic accident, with a complex pelvic injury. Tomography images of crescent fracture dislocation and anterior pelvic injury (**a**–**c**). Fourth year control pelvis radiographs, anterior approach to the sacroiliac joint with two plate–screws (**d**,**e**).

**Figure 3:**
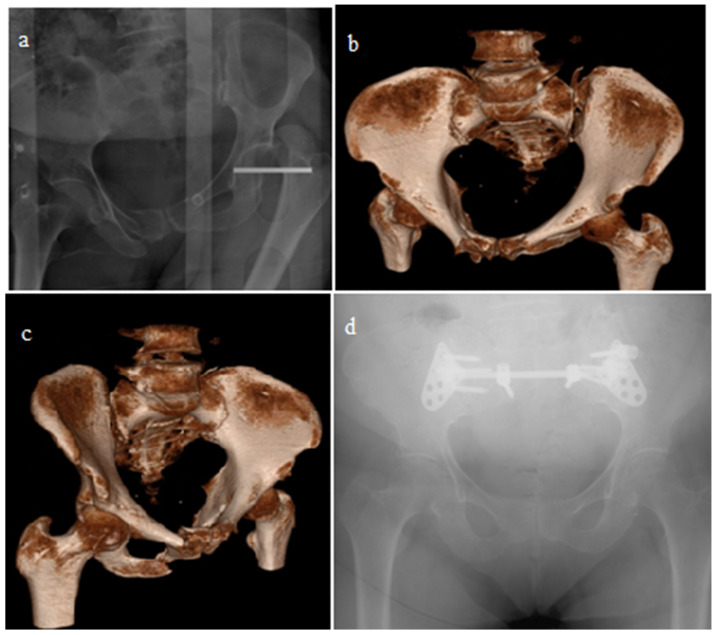
A 37-year-old male patient with bilateral sacroiliac fracture dislocation. Pelvic radiography (**a**) and computed tomography images (**b**,**c**) at the time of injury. Pelvis radiograph at the fifth year of patient follow-up (**d**). Fixation of sacroiliac joints by posterior approach with plate–screws.

Comparisons were made according to the surgeries performed, along with demographic characteristics such as additional injuries and the mechanisms of injury. Patients were treated surgically with open reduction and fixation using plates and screws or closed reduction with percutaneous sacroiliac screws (Figure 1). After open reduction, implants were applied to the sacroiliac joint and/or crescent fractures through anterior or posterior approaches (Figure 2 and Figure 3). The OTA classification was used for fracture classification. In the final follow-up, clinical outcomes were evaluated using a subjective pelvic symptom scoring system [10] (Table 1). The results were classified as excellent, good, fair, and poor, according to the scores received. Additionally, complications such as secondary surgeries, superficial infection, deep infection, and nerve damage (developing postoperatively) were recorded and compared.

### 2.3. Statistical Analysis

Statistical analysis was performed using IBM SPSS Statistics version 26.0 (IBM Corp., Armonk, NY, USA). Descriptive statistics for continuous variables were expressed as the mean ± standard deviation or median (range), while categorical variables were presented as the frequency (n) and percentage (%). The Kolmogorov–Smirnov test was used to assess normal distribution. The Chi-square test and Fisher’s Exact test were used to compare categorical data. A *p*-value < 0.005 was considered statistically significant.

## 3. Results

**Figure 4:**
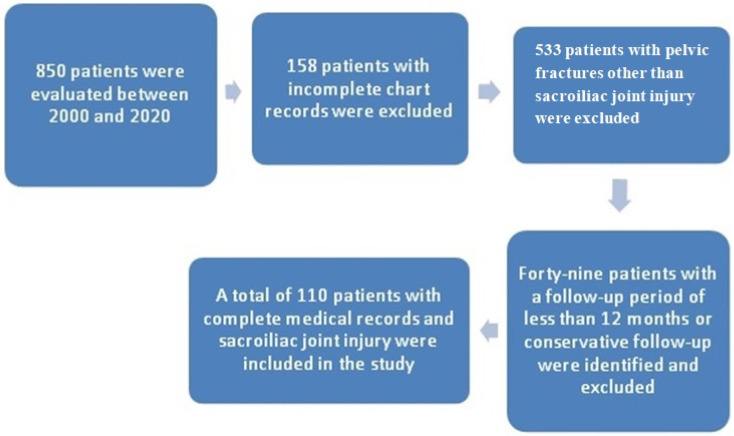
Diagram explaining the inclusion and exclusion of patients.

Surgical interventions were performed on 121 sacroiliac joint injuries involving 110 patients in the study (Figure 4). Eleven patients had bilateral sacroiliac joint injuries and underwent bilateral surgery. The average age of patients at the time of injury was 35.15 years (range from 6 to 80 years). The average follow-up period after the injury was 103.45 months (range from 16 to 253 months). The demographic data of the patients, as well as the injury-related characteristics, are presented in Table 2.

Injuries were more common in males (67.3%), with the most frequent cause being in-vehicle traffic accidents (33.45%). Among the causes of injury, simple incidents such as falls from the same level were particularly observed in elderly patients. Other reasons included crush injuries and animal attacks. The most commonly associated orthopedic injuries of sacroiliac joint injuries and pelvic fractures included radius fractures, found in 13 patients (11.8%) in the upper extremity, and femur fractures, observed in 12 patients (10.9%) in the lower extremity. Additionally, numerous fractures and dislocations in both the upper and lower extremities accompanied pelvic fractures. Extra-extremity injuries were also frequently observed, with the lungs as the most commonly affected organs. Pneumothorax and lung contusions were present in 16 patients (14.55%) alongside sacroiliac joint injuries. Other findings included injuries to the genitourinary system, liver and splenic lacerations, as well as maxillofacial injuries.

In our study of 121 fractures, isolated closed reduction and iliosacral screw fixation were performed in 59 injuries (48.7%) for sacroiliac joint and/or crescent fractures. Thirty-six injuries (29.75%) were openly reduced using an anterior approach and fixed with plates and screws. A posterior approach was employed in 15 injuries (12.4%), with internal fixation using plates and screws. Additionally, open reduction and internal fixation with iliosacral screws were applied in 11 injuries (9.1%).

The subjective pelvic symptom scoring results showed that 52 patients (47%) were rated as excellent, 42 patients (38%) as good, 14 patients (13%) as fair, and 2 patients (2%) as poor. The relationship between the approaches used, the surgeries performed, and the scoring results are presented in Table 3.

Of the 121 injuries, 67 (55.37%) were sacroiliac dislocations, and 54 (44.63%) were crescent fractures. Excellent results were obtained in 32 patients, good results in 20 patients, fair results in 14 patients, and poor results in 1 patient after surgery for sacroiliac dislocations. In the surgeries performed for crescent fracture dislocation, excellent results were found in 25 patients, good results in 21 patients, fair results in 7 patients, and poor results in 2 patients. In the comparison of the surgeries performed for crescent fracture and pure sacroiliac dislocation, there was no statistical difference in the evaluation with functional score according to the injury (*p* = 0.161).

In the evaluation of patients using the subjective pelvic scoring system, excellent results were found in 24 fractures (47.06%) where open reduction and plate–screw fixation were applied, in 25 fractures (42.37%) where closed reduction and iliosacral screws were used, and in 8 fractures (72.7%) where open reduction with iliosacral screws was performed. No superiority was found among the clinical results of the surgeries. The relationship between the functional outcomes of open surgeries with plate–screw internal fixation and those with closed iliosacral screw fixation is presented in Table 4.

In the treatment of fractures, internal fixation of the sacroiliac joint using plate–screw fixation through both anterior and posterior approaches resulted in excellent–good outcomes, reaching around 90% in the evaluation of clinical functions of the fractures. However, there was no significant difference in clinical outcomes based on whether the plate was applied anteriorly or posteriorly (*p* = 0.204). In closed reduction and percutaneous iliosacral screw application, there were relatively more fair–poor results compared to open surgeries; however, this was not statistically significant (*p* = 0.135).

Postoperative complications were observed in thirteen patients (11.8%). Among these, three patients (2.7%) had wound infections, two patients (1.8%) had deep infections, seven patients (6.36%) experienced neurological deficits, and one patient (0.9%) was diagnosed with reflex sympathetic dystrophy in the lower extremity. All deep infections occurred in patients who underwent plate–screw fixation using the posterior approach. Superficial wound infections were found in two patients who underwent plate–screw fixation via the anterior approach, and one was identified in a posterior incision. The implants of the patients with deep infections were removed, and debridement was performed. Superficial infections were managed with antibiotic therapy without surgical drainage.

Postoperative neurological deficits were identified in one patient who underwent plate–screw fixation via the anterior approach and in six patients who received percutaneous iliosacral screws. During follow-up, it was observed that the neurological deficits resolved in three of the patients with percutaneous screws. Although no neurological deficits were detected in surgeries performed with the posterior approach and despite relatively more postoperative neurological complications in the iliosacral screw applications, no significant difference was found when comparing the surgeries (*p* = 0.261).

No patient lost the reduction on the plain X-rays at the final follow-up. During follow-up, the implants of 11 patients (10%) were removed. After open reduction and internal fixation performed via the posterior approach, implants were removed from two patients due to deep infections, with the earliest removal occurring on the 28th postoperative day.

Following closed reduction and percutaneous iliosacral screw application, revision surgeries were performed on two patients after postoperative radiographs and computed tomography images were obtained. In one of the surgeries, the placement of the iliosacral screw was changed due to the incorrect positioning of the screws. In the other revision, open reduction and internal fixation with plates and screws were carried out.

## 4. Discussion

The posterior pelvic structures play a fundamental role in weight-bearing and load transmission to the lower extremities, contributing up to 70% of this function. Therefore, the sacroiliac joints act as a crucial link between the axial skeleton and the lower extremities. Following an injury, anatomical restoration of these joints is critical for optimal functional outcomes, including reduced disability, chronic pain alleviation, and independent mobilization. In many cases, conservative treatment is insufficient to achieve anatomical reduction, necessitating surgical intervention.

Surgical treatment options include minimally invasive closed reduction with iliosacral screws [11] and open surgical techniques such as anterior approach open reduction and internal fixation with plate–screw fixation [12] or posterior approach open reduction and internal fixation [13]. The optimal surgical approach for specific injuries remains a subject of ongoing debate among orthopedic surgeons. Each method presents unique advantages and disadvantages, and the treatment selection is further influenced by patient- and injury-specific factors. For example, anterior access to the sacroiliac joint in obese patients presents challenges in terms of surgical exposure and instrument placement within the confined space. Similarly, in elderly patients where closed reduction is not feasible, open reduction and internal fixation may result in increased bleeding and prolonged operative time.

In our surgical procedures, regardless of whether the etiology was from sacroiliac dislocations or crescent fractures, we initially aimed for appropriate reduction. Patients deemed suitable, based on intraoperative anteroposterior, inlet, and outlet pelvic radiographs, achieved through traction and lateral pelvic compression, underwent closed reduction and percutaneous screw fixation. However, open sacroiliac joint procedures were performed in cases requiring intervention for an associated iliac or acetabular fracture.

Open reduction and internal fixation are the primary treatment modalities for sacroiliac crescent fractures and dislocations [14,15,16]. However, successful fixation using closed reduction and iliosacral screws has also been reported [12,17]. The Day classification system guides treatment of SI crescent fractures and dislocations, providing recommendations on the surgical approach and reduction techniques based on the injury severity [8]. Furthermore, closed reduction and percutaneous screw fixation are often applicable [18]. In our cases of crescent fractures, we utilized iliosacral screw fixation following successful closed reduction. Open surgical intervention was reserved for cases where the reduction was deemed insecure. A study evaluating the safety of iliosacral screw fixation for posterior stabilization in vertically unstable fractures demonstrated this technique to be safe and reported no loss of reduction except in cases involving sacral fractures [19]. Consistent with this study, we observed no loss of fixation during follow-up.

Favorable functional outcomes have been reported following open reduction and internal fixation of the sacroiliac joint. One study of 38 patients reported excellent or good results in up to 70% of cases [20]. Similarly, studies of crescent fractures using both open and closed techniques have demonstrated satisfaction rates as high as 80% [21]. Another study comparing anterior and posterior surgical approaches for SI joint separations and fractures reported good clinical outcomes with both methods [22], although the superiority of the posterior approach for reduction has been suggested [15]. In our study, both approaches achieved success rates of 80% or higher, with no statistically significant difference between them. However, the posterior approach showed a higher incidence of deep infection and abscess formation, sometimes necessitating implant removal. The posterior approach’s proximity to the skin may increase the risk of implant irritation, a potential disadvantage, and implant irritation may be more common with posterior approaches than anterior approaches. The risk of iatrogenic nerve injury is comparatively lower with posterior approaches than with anterior open or percutaneous techniques, and the posterior approach offers superior surgical visualization, exposure, and fixation compared to the ilioinguinal approach via a lateral window, potentially making it easier and more advantageous.

While open surgical techniques offer the advantages of direct visualization and assessment of the joint and fracture and confirmation of reduction, they are associated with significant drawbacks, including prolonged operative and hospitalization times, an increased risk of wound infection, and intraoperative bleeding. These factors are particularly relevant in elderly patients with multiple injuries and contributed to the decision for implant removal in some cases.

A study comparing closed reduction with iliosacral screws and open reduction with plate–screw fixation found that closed reduction was associated with superior functional outcomes [23]. Another study comparing these two methods reported similar success rates for both techniques [24]. Iliosacral screw fixation has been associated with high patient satisfaction rates [25,26]. A study reporting three-year follow-up data on patients treated with closed reduction and percutaneous iliosacral screws demonstrated a 90% success rate [27]. In our patient cohort, a mean satisfaction rate of 85% was observed. In the anterior approach group, we achieved over 90% excellent and good results, which were comparable to those in previous studies. In the closed reduction group, we had excellent and good results of around 80%. There was no statistically significant difference. However, this may be due to the sample size.

Percutaneous iliosacral screw fixation offers several advantages over open surgical techniques, but it also presents unique challenges. Intraoperative fluoroscopic imaging, while allowing for the assessment of reduction, increases the radiation exposure for both the patient and surgical team and carries a risk of misfixation [28]. Factors such as patient obesity, inadequate bowel preparation, and limitations in imaging capabilities (e.g., lack of an appropriate operating room or surgical table for optimal pelvic radiography) can contribute to inaccurate screw placement, and incorrect screw placement can result in injury to the S1–S2 nerve roots or the gluteal artery and nerve [29,30]. Consequently, percutaneous screw placement requires significant experience and a substantial learning curve [28,31].

Conversely, the minimally invasive nature of percutaneous screw fixation is associated with reduced postoperative bleeding, pain, and infection rates, leading to shorter hospital stays [32,33]. In our patient cohort, no postoperative infections were observed following iliosacral screw placement. While bleeding was not quantitatively assessed, no patients required drainage. Neurological deficits were noted in six patients, and two patients exhibited postoperative screw malposition. To mitigate these complications, the use of computed tomography-assisted navigation systems for screw placement may be beneficial [34]. In addition, three-dimensional printing technologies can be used for a better understanding of the fractures, preoperative planning, and patient-specific application guides [35,36,37]. Using these technologies reduces surgical time, blood loss, and complications and improves surgical success and the functional results [35].

This study has several limitations. First, its retrospective design and single-center nature may limit the external validity of the findings. Second, the inclusion of patients with extensive surgical histories introduces potential confounding factors. The absence of complete electronic records and postoperative computed tomography scans for some patients introduced bias into the quantitative radiological measurements, precluding the reporting of quantitative radiological data. Third, the functional outcome assessment focused primarily on relatively simple measures such as patient satisfaction, pain levels, and gait. The heterogeneity of patient demographics and the presence of concomitant injuries prevented the use of standardized outcome scoring systems.

## 5. Conclusions

In conclusion, this study presents a nine-year follow-up of patients treated surgically for sacroiliac joint dislocations and crescent fractures—challenging injuries often resulting from high-energy trauma and associated with significant disruption to pelvic stability. Our findings demonstrate that both open and percutaneous fixation techniques can yield favorable long-term functional outcomes when the anatomical reduction is achieved, with success rates exceeding 80%. While open reduction enables direct visualization and precise correction, it is associated with an increased risk of infection and soft-tissue complications. In contrast, percutaneous fixation offers a less invasive alternative with reduced morbidity, though it demands technical precision and surgical experience. Importantly, no single technique proved superior in all cases, highlighting the necessity of individualized surgical planning based on the fracture morphology, reduction feasibility, patient factors, and imaging availability. While open reduction and rigid fixation may allow for early weight-bearing and mobilization, this does not translate to superior long-term outcomes compared to closed techniques. Treatment selection should be individualized, considering the injury severity and patient comorbidities.

## Figures and Tables

**Table 1:** Subjective pelvic symptom scoring.

Pain at Rest Pain with Activity Limping	None	Mild	Moderate	Severe	Continually
**Score**	1	2	3	4	5
**Treatment** **satisfaction**	Very satisfied	Satisfied	Satisfied, sometimes restrictive	Not satisfied, no activity restrictions	Not satisfied, have activity restrictions
**Score**	1	2	3	4	5

**Minor symptoms:** scores for subjective responses are 1, 2, or 3. **Major symptoms:** scores for subjective responses are 4 or 5. **Excellent:** one minor symptom or no minor symptoms with a satisfaction score of 1–2. **Good:** two or three minor symptoms with a satisfaction score of 1–2 or 3. **Moderate:** with or without minor symptoms; a major symptom with a satisfaction score of 1–2 or 3. **Poor:** any major symptom with a satisfaction score of 4 or 5.

**Table 2:** Demographic data and injury-related characteristics of the patients.

	n	%
**Gender**		
Male	74	67.3
Female	36	32.7
**Etiology of Injury**		
In-vehicle traffic accident	39	33.45
Non-vehicle traffic accident	31	28.2
Fall from a height	22	20
Other	18	16.4
**Additional orthopedic injury**		
Positive	53	48.2
None	57	51.8
**Additional injuries**		
Positive	45	40.9
None	65	59.1
**Fracture Classification (OTA)**		
B1	3	27
B2	56	50.9
B3	11	10
C1	21	19.1
C2	10	9.1
C3	9	8.2
**Sacroiliac joint surgery**		
Closed reduction + ISS	59	48.7
Open reduction + Anterior PS	36	29.75
Open reduction + Posterior PS	15	12.4
Open reduction + ISS	11	9.1
**Complications**	13	11.8
Wound infection		
Superficial	3	2.7
Deep	2	1.8
Neurological deficit	7	6.36
Reflex sympathetic dystrophy	1	0.9

ISS: iliosacral screw operations; PS: plate–screw operations.

**Table 3:** The relationship between the surgeries performed and the scoring.

	Open Reduction + PS n = 51	Closed Reduction + ISS n = 59	Open Reduction + ISS n = 11	*p* Value
**Excellent**	24 (47.06%)	25 (42.37%)	8 (72.7%)	0.204
**Good/Fair/Poor**	27(52.94%)	34 (57.63%)	3 (27.3%)	

PS: plate and screw; ISS: iliosacral screw.

**Table 4:** The relationship between open surgeries themselves, as well as between these and closed surgery and functional outcomes.

	Excellent	Good	Fair/Poor	*p* Value
**Open reduction+ PS** **Anterior approach** **(n = 36)**	19 (52.8%)	14 (38.9%)	3 (8.3%)	0.204
**Open reduction +PS** **Posterior approach** **(n = 15)**	5 (33.3%)	8 (53.3%)	2 (13.3%)	
**Open reduction+ PS** **Anterior approach** **(n = 36)**	19 (52.8%)	14 (38.9%)	3 (8.3%)	0.135
**Closed reduction +ISS (n = 59)**	25 (42.4%)	24 (40.7%)	10 (16.95%)	
**Open reduction +PS** **Posterior approach** **(n = 15)**	5 (33.3%)	8 (53.3%)	2 (13.3%)	0.880
**Closed reduction +ISS (n = 59)**	25 (42.4%)	24 (40.7%)	10 (16.95%)	

PS: plate and screw; ISS: iliosacral screw.

## Data Availability

The datasets generated and analyzed during the current study are not publicly available due to institutional privacy policies but are available from the corresponding author upon reasonable request.
